# Synaptic Inputs to OFF Parasol Ganglion Cells in Macaque Retina: An Analysis Using Serial Blockface Scanning Electron Microscopy

**DOI:** 10.3390/brainsci16060638

**Published:** 2026-06-15

**Authors:** David W. Marshak, Andrea S. Bordt, Nicole B. Harris, James A. Kuchenbecker, Judith Mosinger Ogilvie, Sara S. Patterson, Jay Neitz

**Affiliations:** 1 Department of Neurobiology and Anatomy, McGovern Medical School, Houston, TX 77030, USA; nicole.b.harris@uth.tmc.edu; 2 Department of Ophthalmology, University of Washington, Seattle, WA 98195, USA; abordt@uw.edu (A.S.B.); jkuchen@uw.edu (J.A.K.); jneitz@uw.edu (J.N.); 3 Department of Biology, Saint Louis University, St. Louis, MO 63103, USA; judith.ogilvie@slu.edu; 4 Department Neuroscience, Medical Center, University of Rochester, Rochester, NY 14609, USA; spatte16@ur.rochester.edu

**Keywords:** magnocellular, amacrine cell, bipolar cell, interneuron, primate, electron microscopy

## Abstract

Background/Objectives: Ganglion cells are the projection neurons of the retina, and there are multiple types that differ in their morphology, light responses and central projections. Parasol cells are one of the major retinal ganglion cell types in primates. The presynaptic bipolar cells have been well-characterized, but less is known about the amacrine cells that provide the majority of their inputs. The goal of this study was to identify the amacrine cells presynaptic to the OFF subtype of parasol cells. Methods: Central retinal tissue from an adult macaque was processed for serial block-face scanning EM, and a volume of images of the inner retina located 2 mm temporal to the center of the fovea was analyzed. Results: All the OFF parasol cells in the volume were reconstructed. All the synaptic inputs of two OFF parasol cells were analyzed. They received 80% or more of their input from amacrine cells and the remainder from bipolar cells, almost entirely from the Off diffuse type. Many of the presynaptic amacrine cells were reconstructed sufficiently to be classified as wide-field or narrow-field, and the latter type predominated. Five specific types of presynaptic amacrine cells were identified as AII, A4, knotty bistratified type 1, A13 and wiry type 1. Notably, the same types of amacrine cells are also presynaptic to OFF midget ganglion cells, another major type. Conclusions: These findings suggest that differences between the light responses of midget and parasol ganglion cells likely arise from differences in the presynaptic bipolar cell types.

## 1. Introduction

In primates, parasol ganglion cells have relatively transient responses to many types of light stimuli, and they are particularly sensitive to moving stimuli (reviewed by [1]). The ON type ramifies in the inner half of the inner plexiform layer (IPL), and the OFF type ramifies in the outer half of the IPL. Both types have somas in the ganglion cell layer (GCL) and project to the magnocellular layers of the dorsal lateral geniculate nucleus and the superior colliculus. The dendritic arbors of parasol cells are large and overlap with those of their neighbors (reviewed by [2]). Together, the two types of parasol cells comprise 12% of the retinal ganglion cells in the parafovea and 10% in more peripheral macaque retina. Most of the others are ON and OFF midget ganglion cells [3].

It is particularly important to identify the synaptic inputs to parasol cells in macaque retina because they contain immunoreactive POU6F2, a POU-domain transcription factor that is a risk factor for glaucoma [4]. Parasol cell dendrites are highly sensitive to increases in intraocular pressure (IOP), a condition often associated with glaucoma. In rhesus macaques, elevated IOP affects both major types of ganglion cells, but the dendrites of parasol cells are affected earlier and more severely than those of midget ganglion cells [5]. Parasol cells from macaques with high IOP have smaller, simpler dendritic arbors and are less responsive to patterned and high-frequency stimuli [6].

The OFF parasol cells are the focus of this study, and some of the neurons presynaptic to OFF parasol cells have been identified (reviewed by [2]). These include bipolar cells, local circuit neurons with somas in the inner nuclear layer (INL) whose dendrites receive synapses from photoreceptors in the outer plexiform layer and send axons to the IPL. Most bipolar cell inputs to OFF parasol cells are from the diffuse type, which receive input from multiple cones; only a few originate from midget bipolar cells, which receive input from single cones in this region of the retina [7]. The majority of the synaptic input to OFF parasol cells comes from amacrine cells, local circuit neurons with somas in the INL or the GCL and dendrites in the IPL [8]. The goal of this study was to reconstruct presynaptic amacrine cells from serial electron microscopic (EM) images in order to identify them morphologically and predict how each type might contribute to the light responses of OFF parasol cells.

## 2. Materials and Methods

The Tissue Distribution Program at the Washington National Primate Center, Seattle, WA (WaNPRC) provided retinal tissue from a terminally anesthetized, adult male macaque (Macaca nemestrina) under the approved WaNPRC IACUC protocol, Tissue Banking and Distribution Program (#4277-01). Details have been published previously [9].

Central retinal tissue was processed for serial block-face scanning EM using a previously described protocol [10]. Briefly, tissue taken from 2 mm temporal to the center of the fovea was fixed for electron microscopy, stained en bloc with heavy metals, embedded in epoxy resin, and sectioned in the horizontal plane. Images were acquired at a resolution of 7.5 nm/pixel using a Zeiss (White Plains, NY, USA) Sigma VP field emission scanning electron microscope equipped with a 3View system (Gatan, Inc., Pleasanton, CA, USA).

The retinal volume was approximately 200 µm on each side and contained 937 sections, 70 nm each, spanning from the GCL to the INL. The image tiles were reassembled into a cohesive digital volume, and image registration was performed using Nornir (http://nornir.github.io, RRID:SCR_003584, accessed on 9 June 2026). The database is hosted on a server at the University of Washington.

The volume was annotated using the web-based, multiuser Viking software environment (https://websvc.codepharm.net/Software/Viking/Viking-win-Setup.exe, accessed on 9 June 2026) described previously [11]. Neuronal profiles were annotated using circles with the same diameter, and synapses were annotated as lines.

The cell types ramifying in the IPL were reconstructed and identified using ultrastructural and morphological criteria [12,13]. Axon terminals of bipolar cells contained numerous synaptic vesicles and synaptic ribbons. At some synapses, the bipolar cells were clearly presynaptic, but no ribbons were present, as described previously in the human retina [14]. The bipolar cells were classified based on the morphology of their axon terminals [7].

Processes of amacrine cells typically contained synaptic vesicles that were clustered at synapses. They were divided into narrow- and wide-field types based on the lateral extent of their dendrites. The largest narrow-field amacrine cell in macaque retina described by Mariani [15] is the wavy multistratified type 2. In the volume studied here, the diameter of that cell’s dendritic arbor was 130 µm (not illustrated), and amacrine cells with larger arbors than this were classified as wide-field.

Amacrine cell dendritic arbors were characterized as narrowly or broadly stratified based on the percentage of the IPL depth that they occupied. The boundary between the INL and the IPL was designated as 0% depth and the IPL-GCL boundary as 100%. For some descriptions, the IPL was divided into five strata (S), each 20% in depth, with S1 being outermost (sclerad) and S5 being innermost (vitread). Many of the presynaptic amacrine cells, however, could not be reconstructed sufficiently to be identified.

Data analysis and 3D rendering were performed using an open-source Matlab R2023a program SBFSEM-tools (Mathworks, RRID: SCR_001622) https://github.com/neitzlab/sbfsem-tools, accessed on 9 June 2026, RRID: SCR_017350. Image rendering was performed using the RenderApp function. Processes of amacrine cells, dendrites of ganglion cells and axon terminals of bipolar cells were analyzed using the IPLDepth function. Figures were prepared using Adobe Illustrator 30.5.1 (64 bit) and SBFSEM-tools.

## 3. Results

**Figure 1:**
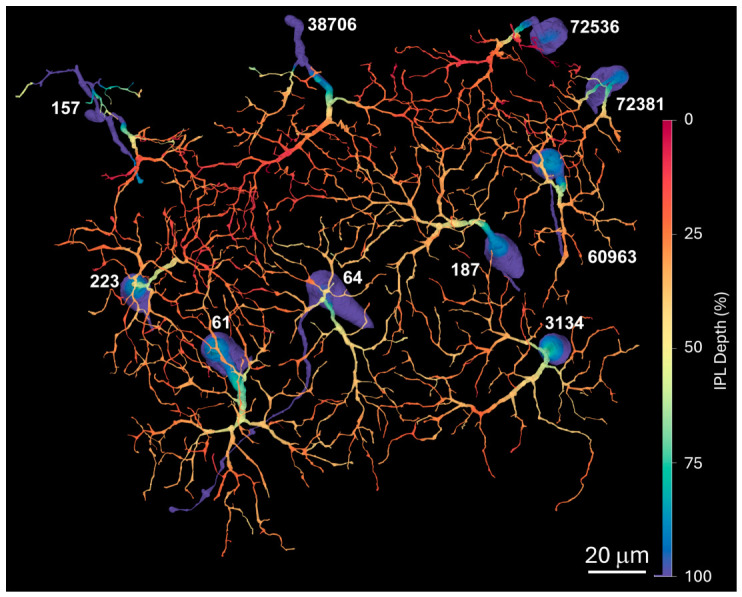
Ten OFF parasol cells were reconstructed, including cell numbers: 61, 64, 157, 187, 223, 3134, 38706, 60963, 72381 and 72536 as they would appear in a horizontal section. The cells in this and subsequent figures were colored by depth in the IPL, with deep red representing 0% depth and deep blue representing 100%. The five OFF parasol cells with complete dendritic arbors were used for the analysis of dendritic overlap. Scale bar = 20 µm.

Ten OFF parasol cells were reconstructed (Figure 1). Their somas were relatively large and located in the outer row of the GCL. Their primary dendrites ascended to S2 of the IPL, where they formed a dense plexus. The average dendritic field diameter of the five OFF parasol cells whose arbors were complete was 85 ± 6.3 µm. The mean dendritic overlap between pairs was 55 µm ± 16.9. The overlap was 65% (55/85), as reported previously [16].

**Figure 2:**
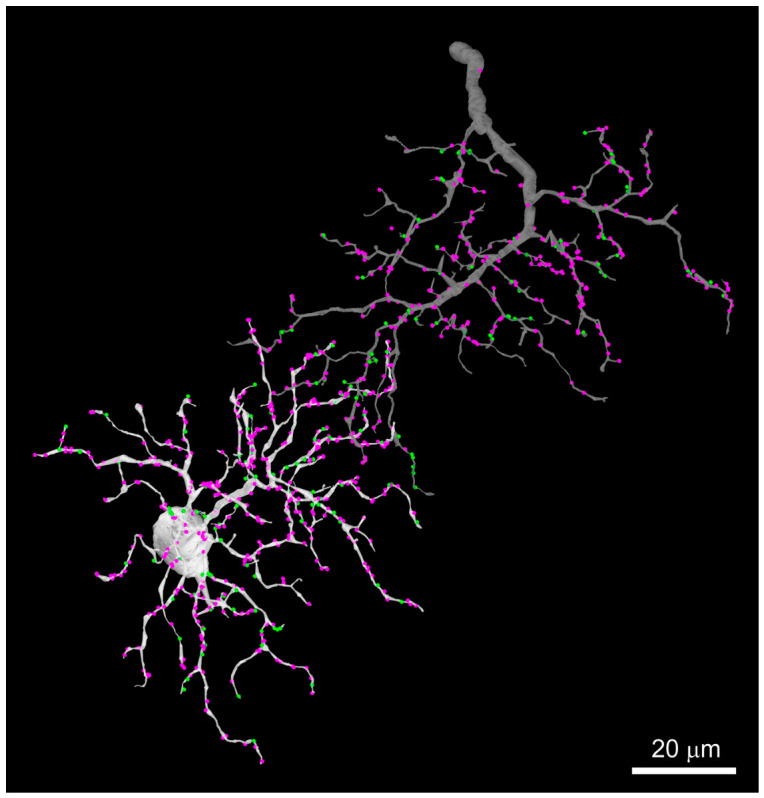
OFF parasol cell 223 (light gray) and 38706 (dark gray) receive synapses (spheres). Bipolar cell synapses are green and amacrine cell synapses are magenta. Note that the synapses were mainly located on distal dendrites and, in some areas, they appeared to be clustered.

The neurons presynaptic to two neighboring OFF parasol cells were reconstructed, and their synapses are illustrated in Figure 2. OFF parasol cell 223 had a complete dendritic arbor and received 508 synapses. Of these, 408 (80%) were from amacrine cells. The vast majority of the other 101 synapses were from OFF diffuse bipolar cells; only three (3%) were from OFF midget bipolar cells.

The dendritic arbor of OFF parasol cell 38706 was incomplete, but it was of particular interest because it received many synapses from a knotty bistratified type 1 (KB1) amacrine cell that resembled those containing immunoreactive vesicular glutamate transporter (vGluT3) [9]. Cell 38706 received 341 synapses. Of these, 283 (83%) were from amacrine cells and the other 58 were from bipolar cells. Of the 50 presynaptic bipolar cells identified, only 5 (10%) were midget bipolar cells.

Many of the amacrine cell processes presynaptic to the two OFF parasol cells were either dendrites or axons of wide-field amacrine cells; these were considered together. For cell 223, 225 presynaptic amacrine cells were classified, and of these, 88 or 39% were from wide-field cells. For cell 38706, 142 presynaptic amacrine cells were classified, of which 52 (37%) were wide-field cells. The other presynaptic amacrine cells that could be classified were the narrow-field type. A small percentage of the presynaptic amacrine cells were also identified morphologically. These should only be regarded as examples, however, because the majority of presynaptic amacrine cells were not identified as specific types.

**Figure 3:**
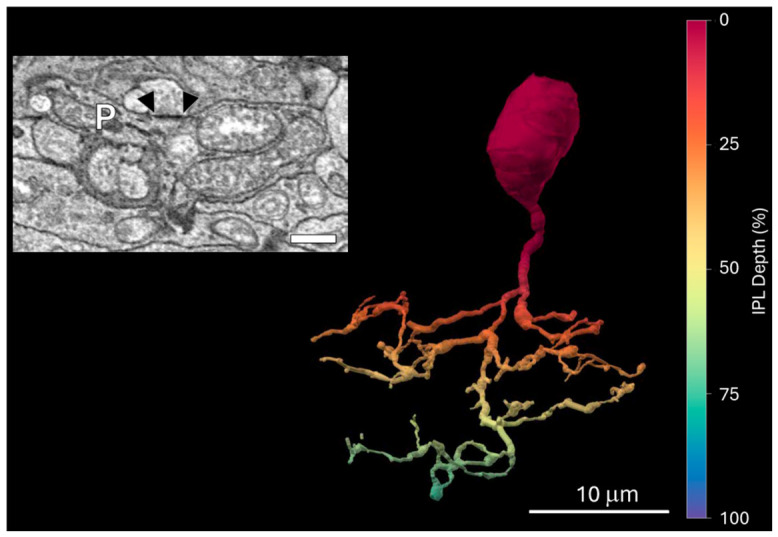
A4 amacrine cell 65376 shown as it would appear in a vertical section. Note the relatively sparse dendritic arbor in the middle three strata of the IPL. Scale bar = 10 µm. Inset: This A4 cell makes a synapse (black arrowheads) onto OFF parasol cell 38706 (P) at location 1569081. Scale bar = 0.5 µm.

Some of the narrow-field amacrine cells presynaptic to the two OFF parasol cells resembled human A4 cells (Figure 3). As described previously using the Golgi method, these had small somas and dendritic arbors. Their recurving dendrites formed a broad plexus in the IPL [17]. Many of the unidentified amacrine cell processes had a similar morphology.

**Figure 4:**
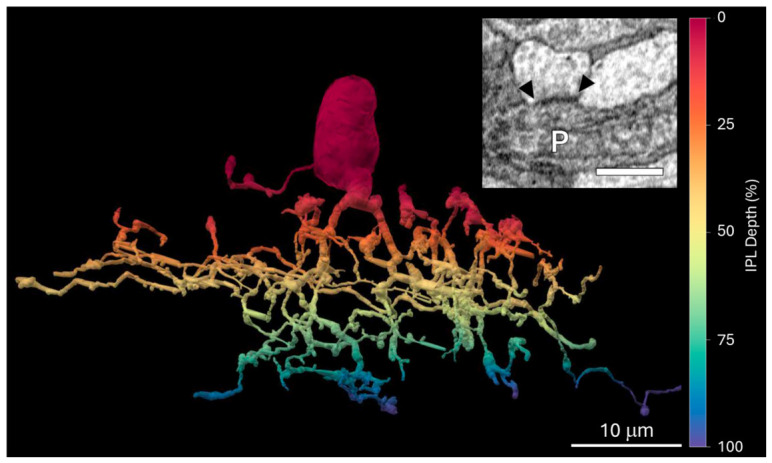
AII amacrine cell 38572 shown as it would appear in a vertical section. Note that the outermost dendrites have lobular appendages and that the dendrites ramify throughout the IPL. Scale bar = 10 µm. Inset: this AII cell makes a synapse (black arrowheads) onto OFF parasol cell 223 (P) at location 1018267. Scale bar = 0.5 µm.

Other narrow-field amacrine cells presynaptic to the two OFF parasol cells were identified as the AII type by their lobular dendritic appendages in S1 and dendrites that ramified throughout the IPL [15,17,18,19]. Their identity was confirmed by the presence of synapses from rod bipolar cells in S5. An example is shown in Figure 4.

**Figure 5:**
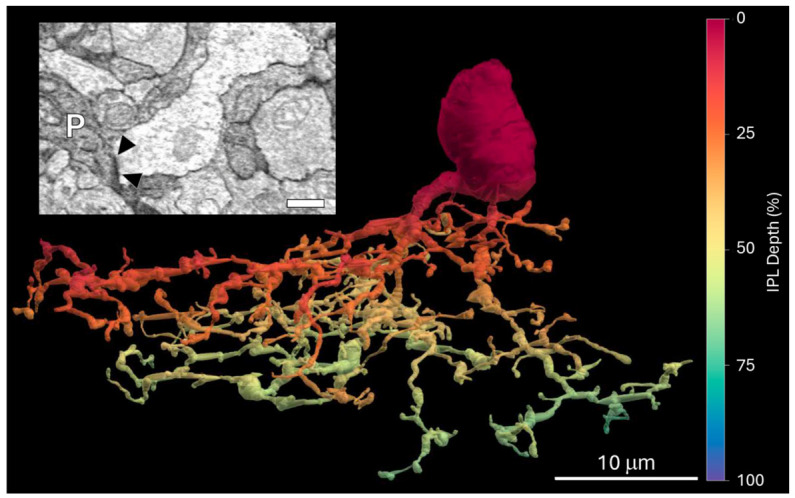
Knotty bistratified type 1 amacrine cell 43016 shown as it would appear in a vertical section. Note the two broad dendritic arbors centered at 23% and 46% depth in the IPL. Scale bar = 10 µm. Inset: this cell makes a synapse (black arrowheads) onto OFF parasol cell 38706 (P) at location 48615. Scale bar = 0.5 µm.

A third presynaptic narrow-field amacrine cell closely resembled the KB1 amacrine cells in the macaque retina [15] and also the amacrine cells in the baboon retina that contain immunoreactive vGluT3 and frequently make synapses onto OFF parasol cells [20]. One of these KB1 cells and many of its pre- and postsynaptic cells were reconstructed completely [9]. Their dendrites formed two dense, relatively broad plexuses on either side of the center of the IPL (Figure 5).

**Figure 6:**
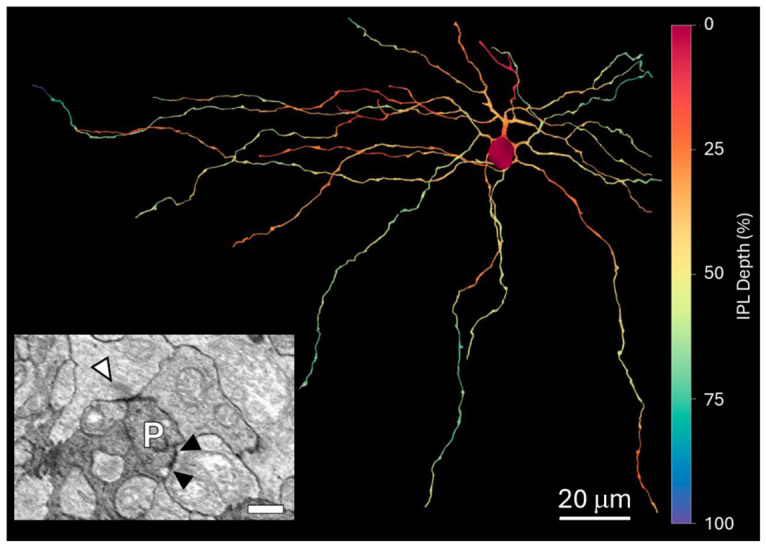
A13 amacrine cell 13883 shown as it would appear in a horizontal section. Note that the dendrites extend from 0 to 80% depth in the IPL. Scale bar = 20 µm. Inset: This amacrine cell forms a synapse (black arrowheads) onto OFF parasol cell 38706 (P), which also receives a synapse from an OFF diffuse bipolar cell (white arrowhead). Scale bar = 0.5 µm.

**Figure 7:**
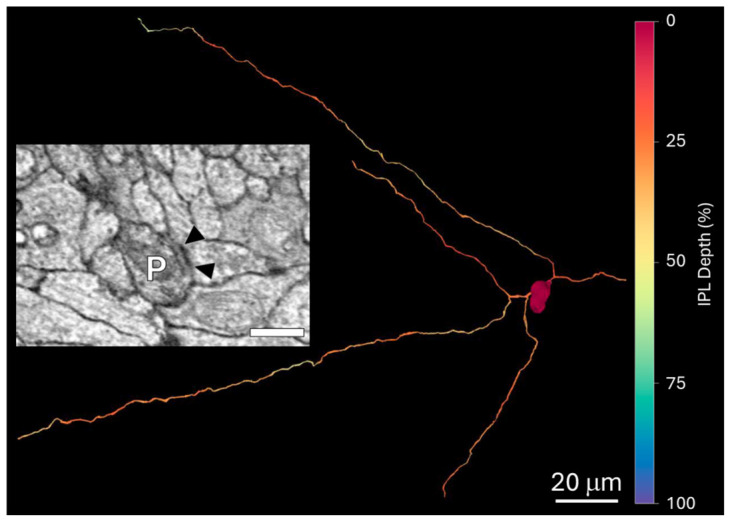
Wiry type 1 amacrine cell 49293 shown as it would appear in a horizontal section. Note the thin, varicose dendrites that ramify in S2 of the IPL. Scale bar = 20 µm. Inset: This amacrine cell makes a synapse (black arrowheads) onto OFF parasol cell 38706 (P). Scale bar = 0.5 µm.

Presynaptic wide-field amacrine cells included an A13 amacrine cell (Figure 6), which was identified by its varicose dendrites that descended to S4 of the IPL [15,17]. Another type of presynaptic wide-field amacrine cell with a relatively small soma and thin, varicose dendrites that ran in the outermost two strata of the IPL was identified as Wiry type 1 (Figure 7). The dendrites branched infrequently at acute angles, as described previously in macaque retina [15,21].

## 4. Discussion

Three of the narrow-field amacrine cells identified in this study are expected to release glycine at synapses onto OFF parasol cells. In the macaque retina, A8 amacrine cells express immunoreactive glycine transporter 1 [22], and AII cells contain immunoreactive glycine [23]. The neurotransmitter of A4 amacrine cells has not been identified, but it is likely that they are also glycinergic, as are most narrow-field amacrine cells in mammals (reviewed by [24]). Glycine is expected to inhibit light responses in the receptive field centers of OFF parasol cells via alpha1 glycine receptors [25]. The light responses from these three types of amacrine cells have not been reported in macaques, but in other mammals, AII amacrine cells have ON responses and A8 amacrine cells have OFF responses [26,27].

The fourth type of narrow-field amacrine cell presynaptic to the OFF parasol cells was the KB1 type, which closely resembles the cells that contain immunoreactive vGluT3 and release glutamate at some of their synapses. OFF parasol cells are among the ganglion cell types that receive input from amacrine cells expressing vGluT3 [20]. There is indirect evidence that these synapses are glutamatergic and excitatory. The gene expression profiles of OFF parasol cells are similar to those of transient OFF alpha cells of mice [28]. OFF alpha cells of mice receive synaptic input from vGluT3 cells [29], and glutamate is the neurotransmitter at synapses from mouse vGluT3 cells to transient OFF alpha ganglion cells [30,31,32].

Wide-field amacrine cells also provided input to the OFF parasol cells, and these typically use the neurotransmitter GABA (reviewed by [24]). OFF parasol cells express GABA_A_ receptors [33], and wide-field amacrine cells are expected to provide tonic, inhibitory input because their conduction velocities are relatively slow and their somas are located at varying distances from the ganglion cells [34,35].

Two types of wide-field amacrine cells presynaptic to OFF parasol cells were identified. Wiry type 1 amacrine cells that ramify in the outer IPL of macaque retina are GABAergic [21]. They have depolarizing responses to decrements in light intensity in their large, narrow receptive field centers which are coextensive with their dendrites, but they have ON-OFF responses to moving stimuli [35]. The light responses of A13 amacrine cells in macaques have not been reported, but in cats, they hyperpolarize to light stimuli and make GABAergic synapses onto OFF retinal ganglion cells [27,36].

Given the large differences between the light responses of OFF parasol cells and OFF midget ganglion cells, it was surprising that they received inputs from many of the same types of amacrine cells [9]. This finding suggests that the differences between the light responses of OFF parasol cells and OFF midget cells are more likely to originate from their bipolar cell inputs. Midget bipolar cells, which receive input from a single cone in this part of the retina, provided the major excitatory input to OFF midget ganglion cells but only a small, variable number of synapses onto the two OFF parasol cells studied most extensively, as reported previously [7].

**Figure 8:**
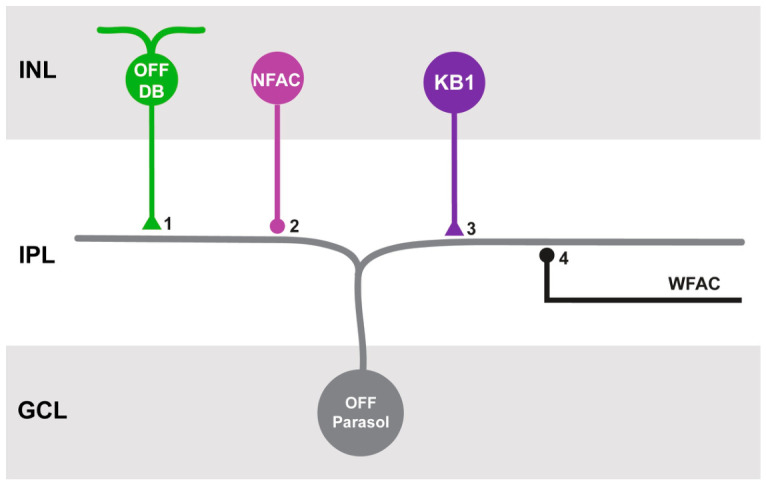
OFF parasol cells receive input from OFF diffuse bipolar cells (OFF DB,1), and these synapses are presumed to be excitatory. The majority of their input comes from various types of amacrine cells, including narrow-field amacrine cells (NFAC, 2), Knotty bistratified type 1 amacrine cells (KB1, 3) and wide-field amacrine cells (WFAC, 4). The wide- and narrow-field amacrine cells are expected to make inhibitory synapses onto OFF parasol cells based on their neurotransmitter content. Because the KB1 cells contain vesicular glutamate transporter 3 and mouse amacrine cells containing this transporter make glutamatergic synapses onto transient OFF alpha ganglion cells, we propose that the synapses from KB1 cells onto OFF parasol cells are excitatory.

The majority of the presynaptic bipolar cells were the diffuse (DB) type. Off midget ganglion cells receive excitatory inputs from OFF midget and DB1 bipolar cells, both of which are expected to have relatively slow, sustained responses to decreases in light intensity based on their membrane properties. The DB2, DB3a, and DB3b bipolar cell types presynaptic to the OFF parasol cells ramify more deeply in the IPL and are expected to exhibit more transient light responses, like those of OFF parasol cells [37]. These findings are summarized in Figure 8.

One caveat is that only a few of the amacrine cells presynaptic to the OFF parasol cells were identified, and it is possible that these unidentified amacrine cells also contribute to the differences between the light responses of OFF parasol and OFF midget ganglion cells. Another potential limitation of this study is that the data came from a single region of only one male macaque retina.

## Data Availability

SBFSEM-tools can be downloaded from GitHub 3.5.5. using the link provided in the text. After selecting Temporal Monkey, the illustrated OFF parasol cells and presynaptic cells can be visualized using the cell numbers from the figure legends. The synapses can be viewed by downloading Viking using the link provided in the text and logging in as “Anonymous_Neitz”, using the password “Anonymous_123” and selecting the Temporal Monkey volume. The synapses illustrated can be viewed using the “Go to location” command.

## Abbreviations

The following abbreviations are used in this manuscript:

GCL	ganglion cell layer
INL	inner nuclear layer
IOP	intraocular pressure
IPL	inner plexiform layer
S	stratum of the IPL
vGluT3	vesicular glutamate transporter 3

## References

[1] Manookin M.B., Patterson S.S., Linehan C.M. (2018). Neural Mechanisms Mediating Motion Sensitivity in Parasol Ganglion Cells of the Primate Retina. Neuron.

[2] Grünert U., Martin P.R. (2021). Morphology, Molecular Characterization, and Connections of Ganglion Cells in Primate Retina. Annu. Rev. Vis. Sci..

[3] Ma I.C.K., Nasir-Ahmad S., Lee S.C.S., Grunert U., Martin P.R. (2023). Contribution of parasol-magnocellular pathway ganglion cells to foveal retina in macaque monkey. Vis. Res..

[4] Lin F., Lin S.T., Wang J., Geisert E.E. (2024). Optimizing retinal ganglion cell nuclear staining for automated cell counting. Exp. Eye Res..

[5] Weber A.J., Kaufman P.L., Hubbard W.C. (1998). Morphology of single ganglion cells in the glaucomatous primate retina. Investig. Ophthalmol. Vis. Sci..

[6] Weber A.J., Harman C.D. (2005). Structure-function relations of parasol cells in the normal and glaucomatous primate retina. Investig. Ophthalmol. Vis. Sci..

[7] Tsukamoto Y., Omi N. (2015). OFF bipolar cells in macaque retina: Type-specific connectivity in the outer and inner synaptic layers. Front. Neuroanat..

[8] Jacoby R.A., Marshak D.W. (2000). Synaptic connections of DB3 diffuse bipolar cell axons in macaque retina. J. Comp. Neurol..

[9] Marshak D.W., Bordt A.S., Yang E.R., Yearick J.N., Mazzaferri M., Kuchenbecker J.A., Ogilvie J.M., Patterson S.S., Neitz J. (2025). Amacrine cell inputs to OFF midget ganglion cells in macaque retina. J. Physiol. Dec..

[10] Patterson S.S., Kuchenbecker J.A., Anderson J.R., Bordt A.S., Marshak D.W., Neitz M., Neitz J. (2019). An S-cone circuit for edge detection in the primate retina. Sci. Rep..

[11] Anderson J.R., Jones B.W., Watt C.B., Shaw M.V., Yang J.H., Demill D., Lauritzen J.S., Lin Y., Rapp K.D., Mastronarde D. (2011). Exploring the retinal connectome. Mol. Vis..

[12] Dowling J.E., Boycott B.B. (1966). Organization of the primate retina: Electron microscopy. Proceedings of the Royal Society of London. Series B, Containing papers of a Biological character. R. Soc..

[13] Grünert U., Martin P.R. (2020). Cell types and cell circuits in human and non-human primate retina. Prog. Retin. Eye Res..

[14] Kolb H., Dekorver L. (1991). Midget ganglion cells of the parafovea of the human retina: A study by electron microscopy and serial section reconstructions. J. Comp. Neurol..

[15] Mariani A.P. (1990). Amacrine cells of the rhesus monkey retina. J. Comp. Neurol..

[16] Dacey D.M., Brace S. (1992). A coupled network for parasol but not midget ganglion cells in the primate retina. Vis. Neurosci..

[17] Kolb H., Linberg K.A., Fisher S.K. (1992). Neurons of the human retina: A Golgi study. J. Comp. Neurol..

[18] Mills S.L., Massey S.C. (1999). AII amacrine cells limit scotopic acuity in central macaque retina: A confocal analysis of calretinin labeling. J. Comp. Neurol..

[19] Wassle H., Grunert U., Chun M.H., Boycott B.B. (1995). The rod pathway of the macaque monkey retina: Identification of AII-amacrine cells with antibodies against calretinin. J. Comp. Neurol..

[20] Marshak D.W., Chuang A.Z., Dolino D.M., Jacoby R.A., Liu W.S., Long Y.E., Sherman M.B., Suh J.M., Vila A., Mills S.L. (2015). Synaptic connections of amacrine cells containing vesicular glutamate transporter 3 in baboon retinas. Vis. Neurosci..

[21] Majumdar S., Wassle H., Jusuf P.R., Haverkamp S. (2008). Mirror-symmetrical populations of wide-field amacrine cells of the macaque monkey retina. J. Comp. Neurol..

[22] Neumann S., Haverkamp S. (2013). Characterization of small-field bistratified amacrine cells in macaque retina labeled by antibodies against synaptotagmin-2. J. Comp. Neurol..

[23] Kolb H., Zhang L., Dekorver L., Cuenca N. (2002). A new look at calretinin-immunoreactive amacrine cell types in the monkey retina. J. Comp. Neurol..

[24] Calbiague-Garcia V., Varro D., Buffet T., Marre O. (2025). The mysterious middlemen making your vision pop: Understanding the function of amacrine cells. J. Physiol..

[25] Grünert U., Ghosh K.K. (1999). Midget and parasol ganglion cells of the primate retina express the alpha1 subunit of the glycine receptor. Vis. Neurosci..

[26] Demb J.B., Singer J.H. (2012). Intrinsic properties and functional circuitry of the AII amacrine cell. Vis. Neurosci..

[27] Kolb H., Nelson R. (1996). Hyperpolarizing, small-field, amacrine cells in cone pathways of cat retina. J. Comp. Neurol..

[28] Hahn J., Monavarfeshani A., Qiao M., Kao A.H., Kolsch Y., Kumar A., Kunze V.P., Rasys A.M., Richardson R., Wekselblatt J.B. (2023). Evolution of neuronal cell classes and types in the vertebrate retina. Nature.

[29] Mani A., Yang X., Zhao T.A., Leyrer M.L., Schreck D., Berson D.M. (2023). A circuit suppressing retinal drive to the optokinetic system during fast image motion. Nat. Commun..

[30] Kim T., Soto F., Kerschensteiner D. (2015). An excitatory amacrine cell detects object motion and provides feature-selective input to ganglion cells in the mouse retina. Elife.

[31] Krishnaswamy A., Yamagata M., Duan X., Hong Y.K., Sanes J.R. (2015). Sidekick 2 directs formation of a retinal circuit that detects differential motion. Nature.

[32] Lee S., Zhang Y., Chen M., Zhou Z.J. (2016). Segregated Glycine-Glutamate Co-transmission from vGluT3 Amacrine Cells to Contrast-Suppressed and Contrast-Enhanced Retinal Circuits. Neuron.

[33] Grünert U., Greferath U., Boycott B.B., Wässle H. (1993). Parasol (P alpha) ganglion-cells of the primate fovea: Immunocytochemical staining with antibodies against GABAA-receptors. Vis. Res..

[34] Greschner M., Heitman A.K., Field G.D., Li P.H., Ahn D., Sher A., Litke A.M., Chichilnisky E.J. (2016). Identification of a Retinal Circuit for Recurrent Suppression Using Indirect Electrical Imaging. Curr. Biol..

[35] Manookin M.B., Puller C., Rieke F., Neitz J., Neitz M. (2015). Distinctive receptive field and physiological properties of a wide-field amacrine cell in the macaque monkey retina. J. Neurophysiol..

[36] Pourcho R.G., Goebel D.J. (1983). Neuronal subpopulations in cat retina which accumulate the GABA agonist, (3H)muscimol: A combined Golgi and autoradiographic study. J. Comp. Neurol..

[37] McLaughlin A.J., Percival K.A., Gayet-Primo J., Puthussery T. (2021). Glycinergic Inhibition Targets Specific Off Cone Bipolar Cells in Primate Retina. eNeuro.

